# Complete chloroplast genome of a subtropical fruit tree *Macadamia ternifolia* (*Proteaceae*)

**DOI:** 10.1080/23802359.2017.1390401

**Published:** 2017-10-17

**Authors:** Jin Liu, Ying-Feng Niu, Shu-Bang Ni, Xi-Yong He, Chao Shi

**Affiliations:** a Yunnan Institute of Tropical Crops, Xishuangbanna, PR China;; b Yunnan Branch of Institute of Medicinal Plant Development, Chinese Academy of Medical Science, Xishuangbanna, PR China;; c Kunming Institute of Botany, Chinese Academy of Sciences, Kunming, PR China

**Keywords:** Chloroplast genome, *Macadamia ternifolia*, *Proteaceae*

## Abstract

*Macadamia ternifolia* is a subtropical fruit tree of the family *Proteaceae*. Chloroplast genome sequences play a significant role in the development of molecular markers in plant phylogenetic and population genetic studies. In this study, we report the complete chloroplast genome sequence of *M. ternifolia*. The chloroplast genome is 159,669 bp long and includes 113 genes. Its LSC, SSC, and IR regions are 88,072, 18,801, and 26,408 bp long, respectively. Sequence comparison of *M. ternifolia* and *M. integrifolia* indicates large sequence conservation between these two species, only few sequence variations including single nucleotide polymorphisms (SNPs) and inserts/deletes (InDels) were detected.

*Macadamia* (*Macadamia* spp.) is a subtropical fruit tree from Australia and is widely cultivated in many other countries for the high oil content of its edible kernel (Mason and McConachie, 1994). There are three major fruit tree species in the genus *Macadamia*: *M. integrifolia*, *M. tetraphylla,* and *M. ternifolia* (Aradhya et al. 1998; Hardner et al. 2009), and *M. ternifolia* is the wild relative of the other two species. Chloroplast genome sequences can provide useful molecular markers for plant genetic studies. A previous study has reported the complete chloroplast genome sequence of *M. integrifolia* (Nock et al. 2014). In this study, we report the complete chloroplast genome of *M. ternifolia*, and hope it would aid further phylogenetic and population genetic studies of the *Macadamia* species.

DNA material was isolated from mature leaves of a *M. ternifolia* plant cultivated in the plant garden of Yunnan Institute of Tropical Crops (YITC), Jinghong, China by using DNeasy Plant Mini Kit (QIAGEN, Hilden, Germany). A specimen of this tree was conserved in YITC. About 10 μg isolated DNA was sent to BGI Shenzhen for library construction and genome sequencing on the Illumina Hiseq 2000 Platform. After genome sequencing, a total of 3.1 Gbp reads in fastq format were obtained and subjected to chloroplast genome assembly. The complete chloroplast genome was annotated with Dual Organelle GenoMe Annotator (DOGMA; Wyman et al. 2004) and submitted to the Genbank (http://www.ncbi.nlm.nih.gov/) under the accession number of MF678834. A physical map of the chloroplast genome was generated by OGDRAW (http://ogdraw.mpimp-golm.mpg.de/) (Lohse et al. 2013).

Our assembly of the *M. ternifolia* resulted in a final sequence of 159,669 bp in length with no gap. The overall A-T content of the chloroplast genome was 61.2%. This chloroplast genome included a typical quadripartite structure with the large single copy (LSC), small single copy (SSC), and inverted repeat (IR) regions of 88,072, 18,801, and 26,408 bp long, respectively. Genome annotation showed 113 full length genes including 79 protein-coding genes, 30 *tRNA* genes, and 4 *rRNA* genes. The genome organization, gene content, and gene relative positions were almost identical to the previously reported *M. integrifolia* chloroplast genome (Nock et al. 2014). Eighteen genes were duplicated in the IR regions. Fifteen genes contained one intron, while three had two introns. Sequence comparison of *M. ternifolia* and *M. integrifolia* indicated large sequence conservation between these two species, only few sequence variations of single nucleotide polymorphisms (SNPs) and inserts/deletes (InDels) were detected.

Phylogenetic tree included *M. ternifolia*, *M. integrifolia,* and the other 11 representative early diverging eudicotyledons species was constructed. Maximum-likelihood (ML) analysis exhibited that *M. integrifolia* clustered with *M. ternifolia*, and other species were highly supported by a bootstrap value of 100 (Figure 1). The evolutionary relationships of these analysed species are consistent with previously reported results (Nock et al. 2014).

**Figure 1.**
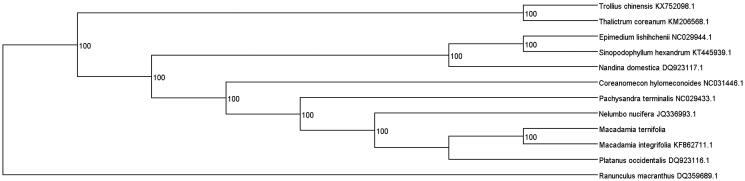
Maximum-likelihood (ML) phylogenetic tree of *M. integrifoia* and the other 11 representative early diverging eudicotyledons species. Number above each node indicates the ML bootstrap support values.

## References

[CIT0001] Aradhya MK, Yee LK, Zee FT, Manshardt RM. 1998 Genetic variability in *Macadamia*. Genet Resour Crop Ev. 45:19–32.

[CIT0002] Hardner CM, Peace C, Lowe AJ, Neal J, Pisanu P, Powell M, Schmidt A, Spain C, Williams K. 2009 Genetic resources and domestication of *Macadamia*. Hortic Rev. 35:1–125.

[CIT0003] Lohse M, Drechsel O, Kahlau S, Bock R. 2013 Organellar GenomeDRAW—a suite of tools for generating physical maps of plastid and mitochondrial genomes and visualizing expression data sets. Nucleic Acids Res. 41:575–581. 23609545 10.1093/nar/gkt289 PMC3692101

[CIT0004] Mason R, McConachie I. 1994 A hard nut to crack: a review of the Australian *Macadamia* nut industry. Food Australia. 46:466.

[CIT0005] Nock CJ, Baten A, King GJ. 2014 Complete chloroplast genome of *Macadamia* integrifolia confirms the position of the Gondwanan early-diverging eudicot family Proteaceae. BMC Genomics. 15:13. 25522147 10.1186/1471-2164-15-S9-S13 PMC4290595

[CIT0006] Wyman SK, Jansen RK, Boore JL. 2004 Automatic annotation of organellar genomes with dogma. Bioinformatics. 20:3252–3255. 15180927 10.1093/bioinformatics/bth352

